# A new species of *Trachymyrmex* (Hymenoptera, Formicidae) fungus-growing ant from the Sierra Madre Oriental of northeastern Mexico

**DOI:** 10.3897/zookeys.706.12539

**Published:** 2017-10-04

**Authors:** Sergio R. Sánchez-Peña, Manuela Citlali Chacón-Cardosa, Ricardo Canales-del-Castillo, Lauren Ward, Diana Resendez-Pérez

**Affiliations:** 1 Departamento de Parasitología, Universidad Autónoma Agraria Antonio Narro, Calzada Antonio Narro 1923, Saltillo, Coahuila, México C.P. 25315; 2 Universidad Autónoma de Nuevo León, Facultad de Ciencias Biológicas, Av. Universidad S/N, Cd. Universitaria, San Nicolás de los Garza, Nuevo León 66455; 3 Department of Entomology, Texas A&M University, TAMU 2475 College Station, TX 77843-2475, USA

**Keywords:** Insecta, Myrmicinae, symbiosis

## Abstract

Here we describe a new species of *Trachymyrmex*, *T. pakawa* **sp. n.**, from the Gran Sierra Plegada range of the Sierra Madre Oriental, in the states of Coahuila and Nuevo Leon, northeastern Mexico. *Trachymyrmex pakawa* is a large-sized species compared to other North American *Trachymyrmex*. Its geographic distribution includes the piedmont of the Gran Sierra Plegada at La Estanzuela, Monterrey, as well as peripheral mountains segregated from the Sierra Madre Oriental (Cerro de las Mitras, Sierra de Zapalinamé, Cañon de San Lorenzo, Cerro de las Letras). The preferred habitats of *T. pakawa* include oak-pine forest at La Estanzuela, xeric oak forest at Zapalinamé and mesic Chihuahuan desert scrub with sotol (*Dasylirion*) at other sites. All localities are on slopes, on very rocky, shallow lithosols overlaying large boulders. This species nests under and between large boulders and rocks. It has not been observed on alluvial or better developed, deeper soils, and it is absent from sites with human activity (urban, disturbed, and landscaped areas). It is closely related to and morphologically similar to *Trachymyrmex smithi*. The known distribution ranges of *T. pakawa* and *T. smithi* almost overlap in Saltillo, Coahuila state. The main character that distinguishes the new species from *T. smithi* is longer antennal scapes in *T. pakawa*; also, different nesting habits (rocky slopes vs. alluvial sites or deep sand in *T. smithi*), and geographic distribution. Phylogenetic analysis of DNA sequences from the mitochondrial marker *cytochrome c oxidase subunit I* (*COI*) and the first intron of the F1 copy of the nuclear protein-coding gene Elongation Factor 1- α *(EF1*-α-*F1)* confirm a sister-species relationship between *T. pakawa* and *T. smithi*. Bayesian coalescent analyses indicate a divergence time of about 8.00 million years before present (95% confidence interval: 4.8–11.5 mya) between *T. pakawa* and *T. smithi*. The divergence of the lineages of *T. pakawa* and *T. smithi* could have been driven by the Pliocene-Holocene desertification of southwestern North America. This process resulted in isolated mesic refugia and forests in the Madrean ranges and piedmonts of northeastern Mexico (the current habitat of *T. pakawa*) while *T. smithi* adapted to the deeper, often sandy soils on the drier desert plains of Coahuila and Chihuahua states in Mexico, and New Mexico and Texas in the USA. Within the Nearctic species of the *Trachymyrmex septentrionalis* species group, *T. pakawa* is the species that is closest (by geographical distribution) to Neotropical species of *Trachymyrmex* like *T. saussurei*.

## Introduction

*Trachymyrmex* Forel is a New World genus of fungus-growing ants closely related to the genera *Atta* and *Acromyrmex*, the well-known leaf-cutting ants (Schultz and Brady 2008). The three genera share similar life-history traits, but *Trachymyrmex* is less derived than the leaf-cutting ants. One major ecological difference between *Trachymyrmex* and the leaf-cutting ants is that *Trachymyrmex* species generally are functional saprotrophs and decomposers; workers usually forage for insect feces, inflorescences, and fallen leaflets as substrate for their symbiotic fungus (Johnson et al. 2006, Sanchez-Peña 2010), while leaf-cutting ants use mainly green, fresh leaves and fresh flowers, harvested from plants, as substrates for cultivation (Weber 1972).

The genus *Trachymyrmex* is considered to have originated in tropical South America (Weber 1972, Mueller and Rabeling 2008); however, there has been at least one large species radiation of the genus in temperate North America, probably related to the Great American Interchange (Bagley and Johnson 2014) or even before the closure of the isthmus of Panama (Philip Ward, pers. comm.). This radiation resulted in about 12 known species that have successfully colonized diverse ecosystems including dry, warm-temperate habitats in the Sonoran and Chihuahuan deserts, and the oak and pine forests of the southeastern and eastern USA, reaching New York state (Weber 1972, Rabeling et al. 2007).

Phylogenetic relationships among the North American species of *Trachymyrmex* and their phylogenetic relationships with the more numerous South American taxa are not clear. Based on analyses of morphological and molecular data for North American species, Rabeling et al. (2007) acknowledged the possible paraphyletic nature of this taxon assemblage. Based on morphological characters and a multilocus phylogenetic analysis, herein we describe a new species of the genus *Trachymyrmex* from limestone-derived montane landscapes in the semiarid Nearctic of northeastern Mexico.

## Materials and methods

### Abbreviation of depositories

**ASU-SIBR** Social Insect Bio Repository, Arizona State University, Tempe, Arizona, USA

**CASC** California Academy of Sciences, San Francisco, California, USA

**UNAM** Colleción Nacional de Insectos, Instituto de Biología, UNAM, Ciudad de México, México

**UAAAN** Departamento de Parasitología, Universidad Autónoma Agraria Antonio Narro, Saltillo, México

**USNM** National Museum of Natural History, Smithsonian Institution, Washington, DC, USA

### Field research

Specimens (workers and queens) were manually collected during daylight at three localities in the Mexican states of Nuevo Leon and Coahuila (Table 1), specifically at the eastern and western edges of the northern Gran Sierra Plegada: south and west of the city of Monterrey, Nuevo Leon, and in the municipality of Saltillo, Coahuila. One nest was excavated at Lomas de Lourdes, Saltillo, Coahuila, on September 3, 2014. Approximately 200 workers, one queen, and a substantial amount of fungus garden were collected.

**Table 1.** *Trachymyrmex* species, accession numbers of *COI* and intron of *EF1*α-*F1* fragment sequences used in the Bayesian phylogenetic and haplotype network analyses, and collection localities of *T. pakawa* and *T. smithi* specimens analyzed.

Species	GenBank, Accession number		Locality
	*COI*	*EF1*-α-*F1* intron	
*T. pakawa_NL1*	MF669548	MF678563	Cerro de las Mitras, Monterrey
*T. pakawa_NL2*	MF669549	MF678564	Estanzuela, Monterrey
*T. pakawa_NL3*	MF669550	Not obtained	Estanzuela, Monterrey
*T. pakawa_COAH1*	MF669551	MF678565	Lomas de Lourdes, Saltillo
*T. pakawa_COAH2*	MF669552	Not obtained	Lomas de Lourdes, Saltillo
*T. smithi T1*	EF539726.1	EF539778.1	Brewster, Texas
*T. smithi T61*	EF539727.1	EF539779.1	La Rosa, Coah. Mexico
*T. smithi T62*	EF539728.1	EF539780.1	La Rosa, Coah. Mexico
*T. smithi T73*	EF539729.1	EF539781.1	La Rosa, Coah. Mexico
*T. smithi T87*	EF539730.1	EF539782.1	El Paso, Texas
*T. smithi T88*	EF539731.1	EF539779.1	Doña Ana, New Mexico
*T. smithi T89*	EF539732.1	EF539784.1	Doña Ana, New Mexico
*T. arizonensis*	EF539739.1	EF539791.1	Cochise Co., Arizona
*T. carinatus*	EF539754.1	EF539806.1	Cochise Co., Arizona
*T. desertorum*	EF539748.1	EF539800.1	Gila Co., Arizona
*T. jamaicensis*	DQ353390.1	EF539829.1	Voucher RA0247, MCZ*
*T. nogalensis*	EF539759.1	EF539811.1	Cochise Co., Arizona
*T. pomonae*	EF539785.1	EF539779.1	Cochise Co., Arizona
*T. septentrionalis*	EU561588.1	EF539813.1	Prattville, Alabama
*T. turrifex*	EU561529.1	EF539815.1	Austin, Texas

* Museum of Comparative Zoology.

**Figure 1.**
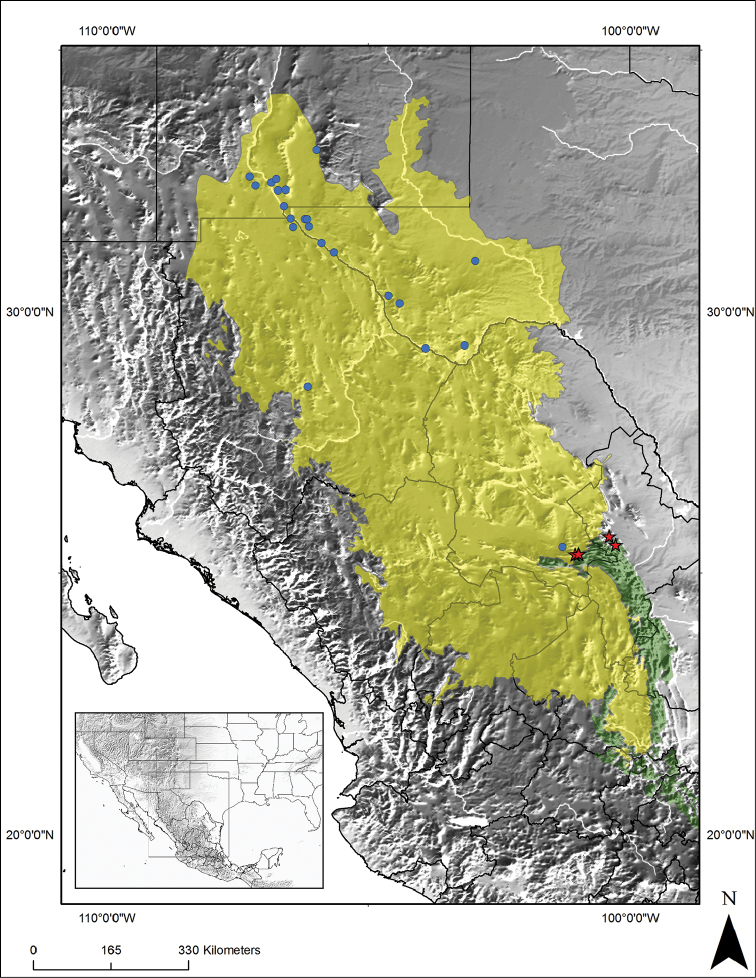
Geographical distribution of *Trachymyrmex pakawa* n. sp. and its closest relative, *Trachymyrmex smithi*, in the southwestern USA and northern Mexico (from Rabeling et al. 2007 and this study). Red stars: collection records of *T. pakawa*. Blue circles: reported collection points of *T. smithi*. Yellow area, Chihuahuan desert. Green area, Sierra Madre Oriental mountain range.

The geographic distribution map (Figure 1) and Table 5 provide detailed information about the localities where *T. pakawa* was collected. A single queen (dealate female) was collected on the soil surface at La Estanzuela, Nuevo Leon, and its measurements are also reported herein.

### Morphological analysis

Morphological characters from field-collected specimens were examined with a SMZ-168TL Motic stereoscope (Motic Inc., Hong Kong) at 50x magnification and greater. Measurements of Head Length (HL), Head Width (HW), Scape Length (SL) and Mesosomal Length (ML) (all in mm) as defined by Rabeling et al. (2007) were obtained using a Mitutoyo Digimatic 500-195-30 digital caliper with a resolution of 0.01 mm under the stereoscope. Cephalic Index (CI) = HW*100/HL and Scape Index (SI) = SL*100/HW were also calculated (Rabeling et al. 2007). Means were calculated from a total of 22 individual workers.

Photographs were taken with a Leica MZ16 APO dissecting microscope (Leica Microsystems, Wetzlar, Germany) using a ProgRes 3008 digital camera mounted on the microscope. Images were taken with the PictureFrame 2.3 software package (Optronics, Goleta, CA). Sequential images were stacked to final 3D form with Helicon Focus software (Helicon Soft, Ltd., Kharkov, Ukraine). Minor corrections (color, background, etc.) were made using Adobe Photoshop CS5 (Adobe Systems, San Jose, CA USA).

### Molecular methods

Initially (in 2010) total genomic DNA was extracted from 1 to 3 worker ants of *T. pakawa* and *T. smithi* following the lithium chloride method (Huang et al. 2000), and *cytochrome oxidase subunit I (COI)* was amplified for initial bioinformatic analysis (below). Subsequently, for both *COI* and Elongation Factor 1-α *(EF1*-α-*F1)* analysis, total DNA was extracted from single worker ants of both species (n = 3 each) using the Pure Link^TM^ Genomic DNA Mini Kit (Invitrogen Carlsbad, CA USA. Identical *COI* sequences were obtained with both methods (see below). Specimens used for analysis were collected at the localities indicated in Table 1.

PCR amplification of the *COI* gene fragment (marker) was carried out (Jacobson et al. 2006) in a volume of 25 ml consisting of 2.5 ml of 10× PCR buffer, 0.75 ml of 50 mM MgCl_2_, 1.25 ml of 2.5 mM dNTPs, 1.0 ml of genomic ant DNA, 1.3 ml of 13.6 mM CI-J2195 primer, 1.0 ml of 15.2 mM Jerry Garcia-CI primer (Jacobson et al. 2006), 0.3 ml of *Taq*DNA polymerase, 5U/ml (all reagents from Invitrogen, Carlsbad CA USA), and 16.9 ml of MilliQ water. PCR amplifications were performed at 95 °C (1 min), 35 cycles of 94 °C (1 min each), 43 °C (1 min), 70 °C (2 min), and 70 °C (2 min). PCR amplification of the nuclear gene fragment of the first intron of the *EF1*-α-*F1* was carried out using the primer pairs U52.1 and L53 as described by Rabeling et al. (2007), in a volume of 25 ml consisting of 2.5 ml of 10X PCR buffer, 0.75 ml of 50 mM MgCl_2_, 0.25 ml of 20 mM dNTP’s, 1.0 ml of genomic ant DNA, 0.5 mM of U52.1 and L53 respectively, 0.3 ml of *Taq* DNA polymerase (5U/ml) (all reagents from Invitrogen, Carlsbad CA USA), and 19.2 ml of MilliQ water. PCR amplifications were performed at 94 °C for 2 min, 35 cycles of denaturation at 94 °C for 30 sec, annealing at 51 °C for 45 sec, extension at 72 °C for 90 sec, and a final extension of 72 °C for 10 min. Amplification products were visualized on 1.2% agarose gel stained with ethidium bromide.

Nucleotide sequencing and purification of PCR fragments were performed at Macrogen Corporation USA (Rockville, MD) using CI-J2195, Jerry Garcia-CI, U52.1, and L53 primers for sequencing.

### Phylogenetic Analysis

The raw sequences were edited in CodonCode aligner (CodonCode Corporation). We included in our dataset previously published sequences; see Table 1 for a complete list of accession numbers of sequences used and the novel ones obtained in this study. Sequences were aligned using CLUSTALW implemented in Mega 6.0 (Tamura et al. 2013). Our final concatenated alignment comprises the partial sequences of the *COI* marker (313 bp) and *EF1*-α (715 bp) marker for a total of 1028 aligned sites and 131 parsimony-informative sites.

We submitted four data blocks to PartitionFinder v1.1.1 (Lanfear et al. 2012) as follows: first, second, and third positions of COI and the intron region of EF1-a-F1. Based on the PartitionFinder analysis under the Bayesian information criterion (BIC) and the “all” search algorithm, our dataset was divided into two partitions: 1) First and second positions of COI and *EF1*-α and 2) third positions of COI. For the first partition the best model was General Time Reversible with invariant sites and a gamma distribution (GTR+I+G) and for the second partition the best model was GTR+G. We conducted a Bayesian phylogenetic analysis employing MrBayes v3.2.5 (Ronquist et al. 2012), with nucmodel= 4by4, nruns = 2, nchains = 4, and sampled freq = 100. Nodes that had posterior probabilities greater than 0.95, were considered well supported. Additionally, a parsimony method to produce a median joining network was used to visualize genetic distance and the geographic associations among *T. pakawa* and *T. smithi* haplotypes using PopArt v.4.6 (http://popart.otago.ac.nz).

Divergence dates between *T. pakawa* and *T. smithi* were estimated using the mitochondrial data with the Bayesian program BEAST v1.4.8 (Drummond and Rambaut, 2007) following the procedure implemented by Seal et al. (2015) for timing the evolutionary events in *T. septentrionalis*. We applied a lognormal relaxed clock, a Yule process tree prior, a UPGMA starting tree, and a TN93 substitution model (Topaliv2.5). The clock model was estimated from a normal distribution with a mean of 0.075 substitutions/sites/lineages/myr. Three independent experiments were performed for 20 million generations and sampled every 2000 generations, with 10% of the trees discarded (burn-in). The output file was evaluated with TRACER v1.4.8 and the effective sample sizes exceeded 200 (Drummond and Rambaut 2007).

The evolutionary distances were computed using the *p*-distance method and represent the proportion of nucleotide changes calculated in MEGA v6 (Tamura et al. 2013). Standard errors of mean *p*-distances among taxa were calculated using 1000 bootstrap replicates.

## Results

### Trachymyrmex pakawa

Taxon classificationAnimaliaHymenopteraFormicidae

Sanchez-Peña, Chacón-Cardosa, Canales-Del Castillo & Reséndez-Pérez sp. n.

http://zoobank.org/9D80EDC0-EF65-4EED-8CA8-D413F120997C

(Figures 2 , 3 , 4)

#### Type material investigated.

**Holotype worker**: MEXICO, Saltillo, Coahuila; Lomas de Lourdes, 25.365181°N, 100.983217°W, 15.viii.2012, dry oak forest, ex ground (S. R. Sanchez-Peña). Collection code: UAN446. Specimen code: USNMENT01125073 (USNM).

#### Paratypes.

Additional specimens (workers) with the same collection information as the holotype deposited at ASU-SIBR, CASC, UAAAN, UNAM and USNM.

#### Additional material.

Additional material: MEXICO, one worker. Cerro de las Mitras, Monterrey, Nuevo León, 25.704834°N, -100.397027°W, 24.viii.2008. Foragers on montane chaparral (S. R. Sanchez-Peña) (UCDC, Philip Ward).

#### Diagnosis (worker).

**Figure 2.**
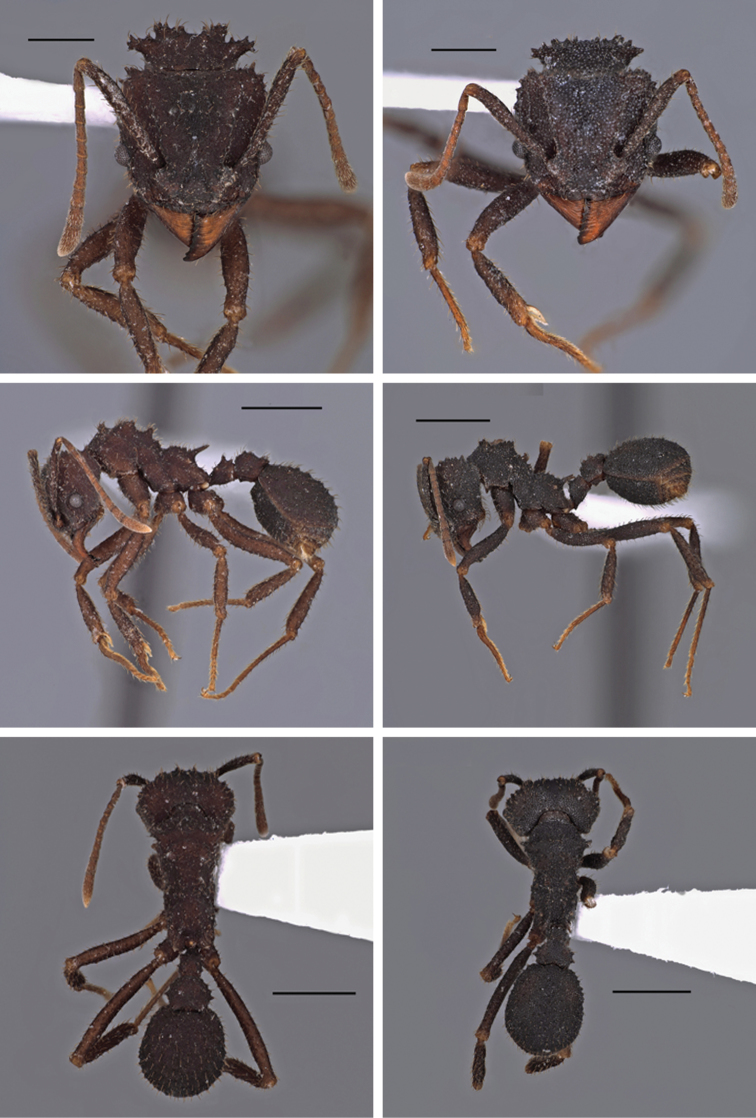
Full face (top row), lateral (middle row) and dorsal (bottom row) views of *Trachymyrmex pakawa* and *Trachymyrmex smithi*. Left column, *T. pakawa*. Right column, *T. smithi*. Scale bars: top, 0.5 mm; middle and bottom, 1.0 mm.

Similar to *Trachymyrmex smithi* but antennal scapes clearly longer, resulting in Scape Index values of 93–109 (Figure 2); head not as clearly cordate, nor broader than long; color dark reddish brown or rarely light ferruginous red. See Measurements in Table 2.

**Table 2.** Dimensions (mm) of morphological features of the worker caste of *Trachymyrmex pakawa* (n = 22) and *Trachymyrmex smithi*

	Head Length (HL)	Head Width (HW)	Cephalic Index (CI) (HW*100/HL)	Scape Length (SL)	Scape Index (SI)(SL* 100/HW)	Mesosomal Length (ML)
WORKER *T. pakawa*	0.975–1.23	1.0-1.375	97.5–116	1.05–1.26	93.5–109.5	1.35–1.71
	x̄ = 1.080	x̄ = 1.115	x̄ = 103.25	x̄ = 1.146	x̄ = 103.00	x̄ = 1.548
WORKER *T. smithi*	0.94–1.25	1.0–1.375	100–111	0.86–1.19	84–89	1.25–1.69

#### Description.

Head trapezoidal, weakly cordate (Figure 2); HW= 1.13, HL= 1.1 (mm) (holotype), nearly square or slightly rectangular, very slightly broader than long; head widest at midpoint between the eye and the posterior corner, and tapering anteriorly. Posterior margin of head is only moderately concave, slightly notched.

Vertex of head and gaster moderately tuberculate, more markedly spinulose than *T. smithi*; tubercles thin and resembling spines, base of tubercles not confluent as in *T. smithi*. Tubercles on preoccipital lobes almost as long as preoccipital spines. Supraocular projections absent. Discal area of mandibles finely striated.

In full-face view, the frontal lobes are small, broadly triangular, usually asymmetrical, with anterior margin longer than posterior (Figure 2).

The margin of the frontal lobes is sub-triangular, smooth, and not crenulated; the base of the frontal lobes lacks projections. Anterior and posterior margin of frontal lobes straight.

Base of antennal scapes lacking lobe. Anterior surface of antennal scapes smooth or weakly microtuberculate. Antennal scapes long, surpassing the posterior corner of head by more than twice their maximum diameter.

Frontal and preocular carina ending separately. In full-face view, frontal carina extends almost to posterior corners, but weakening before reaching vertex. Preocular carina well developed, crossing nearly half distance between eye and frontal carina, curving mesad towards, but not reaching, the frontal carina; frontal carina faintly reaching posterior cephalic margin, forming weakly developed, closed antennal depressions (“scrobes”) without apical tubercles.

Tubercles of gaster and mesosoma small, tubercular setae are weakly to strongly recurved; tubercles on sides of mesosoma minuscule and sparse (Figure 2). Pilosity of gaster and femora consists of hairs only, lacking fine pubescence. Texture of body surface rough, sandpaper-like. In T. *pakawa* the color is dull reddish to (almost always) dark reddish to dark brown, but unlike *T. smithi*, no blackish specimens have been observed. Dorsal projections of mesosoma are multituberculate swellings, tooth- or spine-like (Figure 2). Two median pronotal projections are present, appearing as ridges or multituberculate swellings. The inferior pronotal corner has a rounded, blunt, weakly developed, projecting tooth. Anterior median promesonotal tubercles short, vertical, tooth-like in frontal or posterior view. The anterior mesonotal projections are microscopically multituberculate or multidentate swellings, especially in Saltillo collections; they are nearly as long as pronotal lateral projections in La Estanzuela (Monterrey) but notably longer than pronotal lateral ones in material from Saltillo. Posterior mesonotal projections present, shaped as a ridge or multituberculate tooth or tumulus. Pilosity of mesopleura consisting of approximately twelve short, pale (not reddish) thin curved hairs, about half as long as hairs on mesosomal projections. Projections on inferior and superior margin of mesopleura absent.

The propodeal teeth are strongly divergent, spine-like, and longer than distance separating their bases; the teeth are longer than any promesonotal projections, and longer than projections of basal face. The petiolar node has one pair of teeth in specimens from La Estanzuela, and two (rather clearly) defined pairs of teeth in specimens from Saltillo. Petiolar node from above is as long as broad. Postpetiole from above is distinctly wider than long; the posterior border of postpetiole is notably excised.

#### Etymology.

From the name of an ancient, vanished Native American tribe that used to live in the same general area of arid northeastern Mexico, where *Trachymyrmex pakawa* is known to occur.

#### Distribution.

From warm-temperate forest and scrubland habitats at the northern Sierra Madre Oriental range in the Mexican states of Coahuila and Nuevo León: more specifically, in the northern Gran Sierra Plegada range and mountains between the cities of Monterrey and Saltillo. This species has also been collected in the mountains in the municipality of Iturbide, Nuevo Leon, near coordinates 24.721111, -99.896389, about 100 km to the south of Monterrey.

### Results and discussion: Molecular analysis

We identified two *T. pakawa* haplotypes for each marker (*COI* and *the EF1*-α-*F1*) (Figure 4). A phylogenetic tree was constructed using Bayesian analyses (Figure 4A) of previously published *Trachymyrmex* sequences and sequences of *T. smithi* and *T. pakawa* obtained for this work (Table 1). The *T. smithi* specimens from Ojinaga, Chihuahua, Mexico, yielded *COI* and *EF1*-α-*F1* sequences 100% and 99.99% identical to *T. smithi* Genbank accessions EF539730 and EF539779.1, respectively, from New Mexico, USA; these (New Mexico) and additional *T. smithi* Genbank sequences for both markers were used in our molecular analysis (Table 1). Our phylogenetic tree (fig. 9A) is consistent with the unrooted tree of Rabeling et al. (2007). From the analyses we inferred that *T. pakawa* is a member of the *septentrionalis* species group, and that *T. pakawa* and *T. smithi* are sister species. The *T. pakawa* -*T. smithi* clade is most closely related to the clade consisting of *T. septentrionalis* and *T. pomonae*. These four species are the sister group of a clade consisting of *T. arizonensis*, *T. carinatus*, *T. desertorum*, and *T. nogalensis* (Figure 4); the monophyletic group including these eight species belongs to the *septentrionalis* group (Rabeling et al. 2007). The remaining two species, *T. jamaiciensis* and *T. turrifex*, are more distantly related to the members of the *septentrionalis* group and belong to the *jamaicensis* and *urichii* groups.

Haplotype network maps indicate the genetic distance between *T. smithi* and *T. pakawa* (Figure 4B). For *COI*, there is a difference of > 40 base pairs (bp) between the two species. For the more slowly evolving nuclear *EF1*-α marker the nucleotide difference between the two species is 4 bp.

In our pooled analysis of North American *Trachymyrmex* taxa (Rabeling et al. 2007), the average genetic distance between pairs of species for the *COI* marker without *T. pakawa* is 0.139 (SE = 0.012), whereas the average genetic distance for *COI* between *T. pakawa* and the remaining taxa is comparable: 0.132 (SE = 0.014). The distance between *T. pakawa* and *T. smithi* (0.12) is larger than the distance between *T. smithi–T. arizonensis* (0.11) and *T. smithi–T. carinatus* (0.10). These data support the hypothesis that *T. pakawa* is genetically distinct and reproductively isolated from other *Trachymyrmex* species.

For the *EF1*-α marker, the same patterns are present (although overall distances across all ant taxa considered are much smaller), with the distance between *T. pakawa* and *T. smithi* (0.002) larger than the distance between *T. smithi-T. pomonae* (0.000) (Table 4). But besides the genetic distances, unique insertions and deletions for *T. pakawa* (not shared with the other species) were observed in *EF1*-a.

The pooled analysis for the concatenated *COI-EF1*-a sequences indicated that the average genetic distance between pairs of *Trachymyrmex* species without *T. pakawa* is 0.082 (SE = 0.006), whereas the average distance between *T. pakawa* and the remaining taxa is comparable: 0.066 (SE = 0.005). Also the distance between *T. pakawa* and *T. smithi* (0.04) is similar to the distance between *T. smithi* and other species (0.046–0.054), thus supporting the new species status of *T. pakawa* (Table 4).

**Table 3.** Dimensions (mm) of morphological features of the queen caste of *Trachymyrmex pakawa* (n = 1) and *Trachymyrmex smithi*

	HW	HL	CI= HW*100/HL	SL	SI = SL*100/HW	ML
QUEEN *T. pakawa*	1.28	1.235	103.6	0.99	77.3	1.89
QUEEN *T. smithi*	1.35	1.2	113–114	1.05–1.1	78–79	1.9–2.0

**Table 4.** Genetic distances (for *COI* and *EF1*-α-*F1* marker sequences) between North American species of *Trachymyrmex* including *T. pakawa*. The three datasets are: A) concatenated sequences, *COI* and *EF1*-α-*F1*; B) *COI*; C) *EF1*-α-*F1*.

		*COI -EF1*-α-*F1*								
		1	2	3	4	5	6	7	8	9
**1**	***T. turrifex***									
**2**	***T. jamaicensis***	0.09								
**3**	***T. pomonae***	0.12	0.12							
**4**	***T. arizonensis***	0.13	0.11	0.05						
**5**	***T. desertorum***	0.14	0.13	0.06	0.06					
**6**	***T. carinatus***	0.13	0.13	0.05	0.05	0.04				
**7**	***T. nogalensis***	0.13	0.13	0.05	0.05	0.04	0.03			
**8**	***T. septentrionalis***	0.12	0.12	0.04	0.05	0.07	0.06	0.05		
**9**	***T. smithi***	0.13	0.12	0.05	0.05	0.06	0.05	0.05	0.05	
**10**	***T. pakawa***	0.13	0.12	0.04	0.06	0.06	0.05	0.05	0.04	0.04
		***COI***								
	***T. turrifex***									
	***T. jamaicensis***	0.19								
	***T. pomonae***	0.17	0.14							
	***T. arizonensis***	0.19	0.12	0.11						
	***T. desertorum***	0.22	0.15	0.14	0.12					
	***T. carinatus***	0.17	0.15	0.11	0.11	0.11				
	***T. nogalensis***	0.19	0.16	0.12	0.11	0.11	0.09			
	***T. septentrionalis***	0.17	0.16	0.10	0.11	0.15	0.13	0.11		
	***T. smithi***	0.20	0.15	0.13	0.11	0.13	0.10	0.12	0.15	
	***T. pakawa***	0.19	0.16	0.12	0.14	0.13	0.11	0.11	0.12	0.12
		***EF1***-α-***F1***								
	***T. turrifex***									
	***T. jamaicensis***	0.04								
	***T. pomonae***	0.09	0.10							
	***T. arizonensis***	0.10	0.11	0.02						
	***T. desertorum***	0.11	0.11	0.02	0.03					
	***T. carinatus***	0.10	0.11	0.02	0.02	0.01				
	***T. nogalensis***	0.10	0.11	0.02	0.02	0.01	0.01			
	***T. septentrionalis***	0.10	0.10	0.01	0.02	0.03	0.03	0.02		
	***T. smithi***	0.09	0.10	0.00	0.02	0.02	0.02	0.02	0.01	
	***T. pakawa***	0.10	0.10	0.00	0.02	0.02	0.02	0.02	0.01	0.002

In general, the larger genetic distances among these North American *Trachymyrmex* species and clades are always between [*T. turrifex* or *T. jamaiciensis*] and the remaining taxa including *T. pakawa*.

Both phylogenetic and network analyses showed the same relationships among *T. smithi* and *T. pakawa*. No shared haplotypes were found between *T. smithi* and *T. pakawa* (Figure 4B). For *COI*, there is a difference of 34 base pairs (bp), whereas for *EF1*-α the difference is 3 bp (Figure 4B). Considering the small number of specimens analyzed (five for *COI* and three for *EF1*-a) there is possibly significant genetic variation within *T. pakawa*, since two haplotypes were found for each marker (Figure 4B). Comprehensive analyses are required to elucidate the population structure of *T. pakawa*.

Bayesian coalescent analyses indicate a divergence time of about 8.00 million years before present (95% confidence interval: 4.8–11.5 mya) between *T. pakawa* and *T. smithi*. The divergence of the lineages of *T. pakawa* and *T. smithi* could have been driven by the Pliocene-Holocene desertification of southwestern North America.

## General Discussion and Comments

Among the Nearctic *T. septentrionalis* species group, this species is the closest (by geographical distribution) to Neotropical species, including the related *Trachymyrmex saussurei*. We initially identified these ants as specimens of *T. smithi* (Buren 1944). However, the main differences between *T. pakawa* and *T. smithi* include differences in morphology, geographical distribution, ecology, and habitat preference. Morphologically, the antennal scapes of *T. pakawa* are distinctly longer that those of *T. smithi* throughout its range (Figure 2) resulting in Scape Index values of 93–109, vs. 84–89 in *T. smithi* (Rabeling et al. 2007). This is the most obvious differential trait. Also, the head is not as clearly cordate as in *T. smithi*. The color of workers is dark reddish brown, or rarely light ferruginous red, as opposed to deep dark brown, almost black, in *T. smithi* (Figure 2). In general, *T. pakawa* has a more spinulose appearance than *T. smithi* (Figure 2).

Standard measurements of the queen caste are also provided from one specimen collected at La Estanzuela, Monterrey, NL (Table 3). Morphological measurements for this caste are very similar between *T. pakawa* and *T. smithi* (Table 3).

*Trachymyrmex pakawa* is one of the largest species in size for this genus in North America. Color is almost always dark- brown; only one collection, from La Estanzuela, included rusty workers; but these could have been callow (newly emerged) foragers.

The specimens from La Estanzuela had the longest scapes (0.96–1.26 mm; n = 14, compared to Lomas de Lourdes, Saltillo: 0.98–1.10 mm, n = 6) and Mitras (1.05–1.11, n = 2); however these differences could be influenced by small sample size. In 14% of *T. pakawa* workers, HL is larger than HW (i.e., the head is longer than wide).

Morphologically *T. pakawa* is sharply different from the nearly sympatric *T. turrifex* (which has subparallel preocular and frontal carinae, shorter anntenal scapes, and is lighter in color) and very clearly larger than *T. septentrionalis* (Rabeling et al. 2007), the southern distribution limit of which is about 300 km to the north, near San Antonio, Texas.

Unlike *T. nogalensis, T*. *pakawa* possesses well-developed, long, frontal carinae that extend to the posterior corner of the head; it thus lacks the short, distinctive antennal depressions (so-called “scrobes” in Rabeling et al. 2007) formed by the frontal and preocular carinae. *T. pakawa* has shorter antennal scapes and longer propodeal spines than *T. nogalensis*. It also lacks the proximal narrowing or “waist” of the scape, and the lobe just distal to the narrowing, present in *T. nogalensis*.

The antennal scape in *T. carinatus* is considerably longer than in *T. pakawa*, (SI 117–152 in *T. carinatus* vs. 93.5–109.5, mean = 103.00 (n=22) in *T. pakawa*). In *T. carinatus*, the body is only moderately tuberculate and the color is yellowish brown. *T. pakawa* also lacks the sharp carinae on the vertex of workers, present in *T. carinatus* and indicated by an arrow in figure 3B of Rabeling et al. (2007), and described by Mackay and Mackay (1997).

In *T. pakawa*, the propodeal teeth are long, longer than the space separating their bases. In other species of the *T. septentrionalis* group (i. e. *T. carinatus* and *T. septentrionalis*) the propodeal spines are as long or shorter than the distance between their bases.

**Figure 3.**
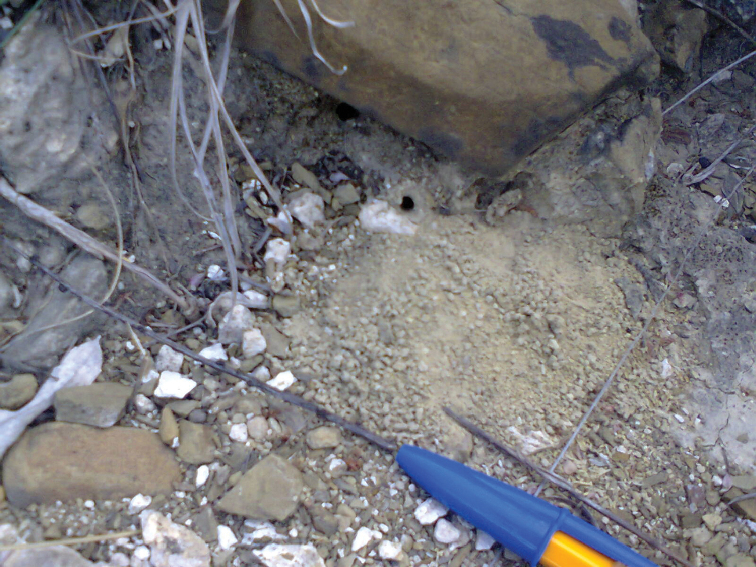
Nest entrance of *T. pakawa*. Cerro de las Letras, Saltillo, Coahuila.

*T. pakawa* and *T. smithi* differ also in their general habitat and nesting preference: *T. pakawa* nests occur in very rugged, sloping rocky terrain (Figure 3), similar to the nests of *T. nogalensis* (Rabeling et al. 2007), whereas *T. smithi* nests are often in deep sandy soils, on open flat areas or gently sloping bajadas (alluvial fans) (Cole 1952, Rabeling et al. 2007, SRSP unpublished observations).

The nests of *T. pakawa* are very inconspicuous compared to the nests of *T. smithi* (Figure 3). Colonies of the latter species can be rather populous, the largest of the species of *Trachymyrmex* that occur in the United States with more than a thousand workers (Rabeling et al. 2007, SRSP unpublished observations), while (from the observation of one excavated nest) we estimate 200–300 workers for mature nests of *T. pakawa* in favorable habitats. *Trachymyrmex pakawa* appears to be restricted to temperate, often semiarid forest and piedmont desert scrub on the mountain ranges and slopes of the calcareous northern Sierra Madre Oriental of northeastern Mexico, while *T. smithi* inhabits many localities within the Chihuahuan desert, from Coahuila to New Mexico (Mackay and Mackay 2002, Rabeling et al. 2007). Most records of *T. smithi* are from igneous sandy soils at the western Chihuahuan desert (Figure 1; Rabeling et al. 2007).

*Trachymyrmex pakawa* nesting habits, in the spaces between and under large or huge rocks on the mountain slopes, are similar to those of the fungus-growing ant *Cyphomyrmex wheeleri* Forel (Wheeler 1907) that is sympatric with the new species at several locations of Saltillo (SRSP unpublished observations).

**Figure 4.**
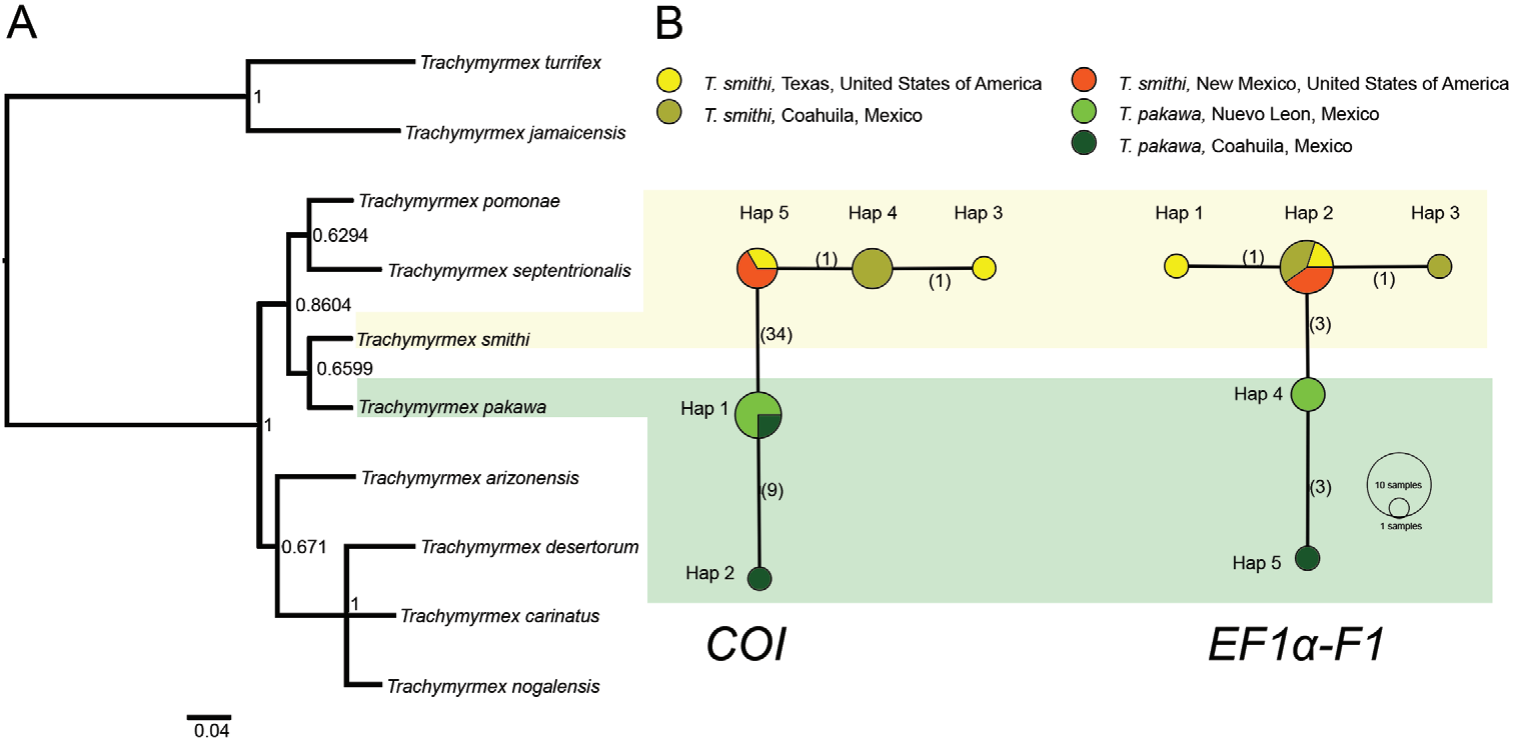
**A** Bayesian phylogenetics estimates and **B** haplotype networks of *COI* and *EF1-α-F1* sequences of North American *Trachymyrmex* species. The phylogenetics inference was calculated with *COI* and *EF1-α-F1*; clade support is indicated above branch posterior probability by Bayesian inference. Due to the incomplete taxon sampling the tree was midpoint rooted. In the minimum-spanning haplotype networks for *COI* and *EF1-α-F1*, each circle represents a haplotype, with size proportional to the haplotype’s frequency in the population. Numbers along branches represent substitutions.

### Modifications to existing identification keys

To include *T. pakawa* we modified the taxonomic identification key to North American *Trachymyrmex* workers (Rabeling et al. 2007) as follows:

**Table:** 

3	Frontal carinae relatively short, not extending towards the posterior corner of the head. Preocular carinae curving strongly to meet the frontal carinae, forming short, distinctive depressions or “scrobes” that end slightly behind the level of the eye (figure [10B] in Rabeling et al. 2007). Antennal scape long (SI 117–152), narrowing abruptly as it approaches the antennal insertion, a small but conspicuous lobe is present just distal to the narrowing (figures 10 and 11 in Rabeling et al. 2007)	***T. nogalensis***
–	Frontal carinae long, extending well past the eye towards the posterior corners of the head. In side view, preocular carinae not joining the frontal carinae (rarely touching the carinae in *T. carinatus*). Antennal scapes short (SI 84–89) or long (SI 94–113), not as long as in *T. nogalensis*. Scape gradually narrowing as it approaches the antennal insertion, lobe as described above absent (figures 1, 3, 5, 6, 12, 13, 15 and 17 in Rabeling et al. 2007)	**4**
4	Combining the following: In full-face view, frontal lobes prominent, shaped uniquely as in figure 1B in Rabeling et al. 2007; the posterior margin of the lobe forming a broad notch where it meets the frontal carinae. Antennal scapes long (worker SI 103–113; queen SI 96–105). Propodeal spines shorter than the distance separating their bases. First gastric tergite strongly tuberculate (figure 1 in Rabeling et al. 2007)	***T. arizonensis***
–	In full-face view, frontal lobes simple, not shaped as above, but rounded or triangular (figure 3B in Rabeling et al. 2007). Antennal scapes short (worker SI 84–89 (105 in Rabeling et al. 2007) or long (SI> 93). Propodeal spines longer or shorter than the distance separating their bases. Gastric tubercles variously developed, sometimes small or nearly absent	**5**
5	Relatively large species (HW 1.0–1.38). In larger workers, head square or clearly broader than long, square or slightly broader than long in smaller workers; in full-face view head often appearing cordate in larger workers (figure 17B in Rabeling et al. 2007); antennal scape short, (SI 84–89) or long (SI 93.5 - 109.5). Propodeal spines longer than the distance separating their bases. Color dark reddish brown, fuscous or black (figure 17 in Rabeling et al. 2007); from typical flat, open Chihuahuan desert habitats, or rocky habitats of the northern Sierra Madre Oriental	**6**
–	Smaller species (HW 0.78–1.12). Head shape variable, usually more or less square, sometimes broader than long. Posterior border weakly to moderately emarginate, but head never appearing cordate in full-face view—even in larger workers (figure [3B] in Rabeling et al. 2007). Propodeal spines variable in length, often as long as or shorter than the distance separating their bases. Color never black, usually not dark reddish brown, commonly brownish yellow to medium reddish-brown	**7**
6	Combining the following: in larger workers head clearly broader than long, slightly broader than long to square in smaller workers; antennal scape short, SI 84–89; in full-face view head appearing cordate in larger workers (figure 17B in Rabeling et al. 2007). Color black to blackish red (figure 17 in Rabeling et al. 2007); from typical flat, open Chihuahuan desert habitats, usually on sandy soil	***T. smithi***
–	Similar to *T. smithi* but antennal scapes clearly longer (this work), SI 93.5–109.5; head not as clearly cordate, nor broader than long; color dark reddish brown (Figure 2, this work) or rarely light ferruginous red, not blackish; from warm-temperate forest and scrubland habitats at the northern Sierra Madre Oriental of Mexico (Gran Sierra Plegada mountains near Monterrey, Saltillo and Iturbide). Nests usually on steep terrain, slopes and near cliffs, on very rocky (not sandy) soils	***T. pakawa***

### Known species range

Specimens of *Trachymyrmex pakawa* were collected in the Mexican states of Nuevo Leon and Coahuila. The known species range extends between the opposite edges (west and east) of the Gran Sierra Plegada section of the northern Sierra Madre Oriental mountain range. The more geographically distant populations observed and sampled at the edges of this mountain range, are separated by about 80 km on a straight line (see coordinates, La Estanzuela and Cerro de las Letras). In between these known extreme distribution points, several populations have been detected (Table 5).

**Table 5.** Collection localities for *Trachymyrmex pakawa*.

Locality	Altitude	Google Earth coordinates
Estanzuela 1, Monterrey, Nuevo Leon	600 m	25.540425°N, -100.272864°W
Estanzuela 2, Monterrey	700 m	25.536932°N, -100.276289°W
Cerro de las Mitras 1 (Pico Apache), Monterrey	900 m	25.704834°N, -100.397027°W
Cerro de las Mitras 2 (Pico Apache) Monterrey	1300 m	25.704166°N, -100.400833°W
UAAAN reforestation edge, Saltillo, Coahuila	1650 m	25.351378°N, -101.041464°W
Cerro de las Letras, Saltillo	1800 m	25.358855°N, -101.049164°W
Lomas de Lourdes spring, Saltillo	1750 m	25.359984°N, -100.980606°W
Lomas de Lourdes dry creek, Saltillo	1700 m	25.365181°N, -100.983217°W
Cañon de San Lorenzo, Saltillo	1900 m	25.330996°N, -100.986779°W

### Habitats

This species inhabits very rocky soils, on moderate to very steep slopes, usually between and under large limestone boulders and rocks with a very thin (10–20 cm) cover of lithosol (limestone derived). This seems the main common aspect to most habitats where *T. pakawa* has been found. The sites are rather diverse montane habitats of the southern Nearctic: gallery forests, oak and oak-pine forests, and xerophilous Chihuahuan scrub on slopes (“submontane scrub”) (Table 4).

With the possible exception of the Lomas de Lourdes (Saltillo) population, all these localities are within stands of lechuguilla (*Agave lechuguilla*), or within 300 m or less from rocky outcrops where this plant is present. Even the gallery forest at La Estanzuela is within a few hundred meters from lechuguilla stands. This underscores the xerophilous nature of *T. pakawa*.

### Description of localities where *T. pakawa* has been collected

***Coahuila state***

1. We collected *T. pakawa* in August of 2012–2015, above the Lomas de Lourdes area of Saltillo, on the slopes of the Sierra de Zapalinamé, a small isolated mountain range separated from the main Sierra Madre range by narrow, arid valleys. 25.35998°N, -100.98060°W. Altitude is 1750 m. The ant was also collected at 25.365181°N, -100.983217°W, at an altitude of 1700. Vegetation at these sites is a xerophilous forest of small oaks (4–5 m tall) (*Quercus laeta* and the endemic *Q. saltillensis*), mountain mahogany (*Cercocarpus* spp.), antelope bush (*Purshia plicata*), weeping juniper (*Juniperus flaccida*), and madrone (*Arbutus xalapensis)*. The canopy is rather dense; the ants were observed mainly on exposed slopes. Annual precipitation here is 400 mm, with abundant fog spells. On this western (rain shadow) side of the Sierra Madre Oriental, the soil is granular, and usually covered with a thick, but usually dry, litter layer of oak leaves. The ants live in the more mesic creeks and microhabitats of the range (NW slope) in an area with a few very small, intermittent springs, indicating a shallow water table. As elsewhere for *T. pakawa*, this population is not abundant; only five colonies and one nest entrance have been observed despite active searching over several years. The ants nest in very shallow lithosol covering a rocky layer, or among large limestone boulders (outcrops); the nests are possibly at least one meter deep in order to reach moisture pockets in spaces underneath the boulder layer. The tridimensional structure of aquifers is complex on these slopes, and nesting location might be influenced by the water table.

2. Cañon de San Lorenzo, Sierra de Zapalinamé. 3 August 2013. 25.330996°N, -100.986779°W; altitude is 1900 m. This site is at about 5 km (south) from the previous location. Foraging workers were collected on a sun-exposed limestone peak (western slope) covered with xerophilous scrub (*A. lechuguilla*), sotol (*Dasylirion* sp.), and *Yucca filifera*; above and at short distance from a mesic creek with Arizona cypress, (*Cupressus arizonica*). The foragers were walking among boulders. The site is a generally arid landscape with very small, scattered springs.

3. Reforestation edge, Universidad Autónoma Agraria Antonio Narro (UAAAN) fields, located at 5 km from the Cañon de San Lorenzo population. 10 October 2013. 25.351378°N, -101.041464°W. Altitude is 1650 m. The habitat is Chihuahuan desert; the observed nest was under low scrub of *Acacia greggii* (uña de gato, cat´s claw) at the edge of an area reforested with exotic pines (*Pinus halepensis*).

4. Cerro de las Letras, west of UAAAN campus. 10 September 2012. 25.35885°N, -101.04916°W; altitude is 1750 m. This site is on the steep eastern slope of a hill; the vegetation consists of Chihuahuan desert scrub with sotol, cacti (*Opuntia* spp.), *Tecoma stans* (yellow bells) and *Yucca* sp. The site is right above the creosote bush (*Larrea tridentata*) life zone. At this location (and probably others), *T. pakawa* is sympatric with the cryptic, highly xerophilous fungus-growing ant, *Cyphomyrmex wheeleri* (SRSP unpublished observations). One nest entrance of *Trachymyrmex pakawa* was also observed here, on a very small flat area on rocky outcrop; it had a small pile (a 4 cm fan) of excavated, lighter soil particles on one side of the entrance (Figure 3).

***Nuevo Leon state***

5. La Estanzuela creek (a state park of Nuevo Leon), on the southern edge of the city of Monterrey. 8 August 2009. 25.54042°N,-100.27286°W, 600 m altitude. This location is along an intermittent stream that flows down from the Sierra Madre into the Rio La Silla, which is part of the Rio Grande Basin. This site is on the eastern slope (not rain shadow side) of the Sierra. The locality is a warm-temperate gallery forest of sycamore (*Platanus mexicana*), Monterrey oak, *Quercus polymorpha*, and other *Quercus* species. *Trachymyrmex pakawa* forages at a few meters from the pebbly riverbed, on loam-clay reddish soil of variable depth with interspersed large rocks. The surrounding habitat within a short distance (sometimes 200 m) from the stream is clearly xerophilus: on sun-exposed boulders, there is a thorny leguminous scrub with *Agave lechuguilla*, *Acacia* spp., *Caesalpinia mexicana*, *Cordia boissieri*, and Cactaceae. Annual precipitation is 500–700 mm; annual temperatures range between 5–40° C. We collected *T. pakawa* here repeatedly from 2007–2013.

6. Upland and up the stream at La Estanzuela, at the previous location. 15 August 2012. 25.536932°N, -100.276289°W; 700 m altitude. This site is an ecotone of oak forest (*Quercus rysophylla*, *Quercus polymorpha*)-pine forest (*Pinus pseudostrobus*), over a limestone cliff. A very shallow, reddish litosol between boulders covers the bedrock. A nest entrance was observed there on deeper soil on a slope. The entrance was an inconspicuous hole (2–3 mm diameter) on a flat, sloped area on loamy soil. No accumulations of soil or detritus were observed at this nest entrance, probably due to the slope and rain.

7. Pico Apache (a peak at Cerro de las Mitras mountain) Monterrey, Nuevo León. 24 July 2008. 25.704834°N, -100.397027°W; 950 m altitude. This site is on the NE slope of the mountain, in Chihuahuan desert scrub/”submontane” scrub, with presence of *Agave lechuguilla* and sotol (*Dasylirion* sp.) scrub among boulders.

8. Pico Apache, Cerro de las Mitras, Monterrey, Nuevo León. 24 July 2008. 25.704166°N, -100.400833°W; 1300 m altitude. The vegetation at this site is scrub with sotol, *Agave bracteosa* (squid agave), *A. lechuguilla*, *Opuntia stricta*, *Calia secundiflora* (mescalbean or frijolillo), and short oaks, up to 2.5 m tall (*Quercus* sp.). Both locations at Cerro de las Mitras are moister than the surrounding plains.

### Ecological observations

Unlike *T. smithi*, its closest relative, *T. pakawa* has very inconspicuous nest entrances that are rather hard to find (Figure 3). There is no mound, crater, or turret as in other attines like *Trachymyrmex turrifex* or *Mycetosoritis hartmanni* (Wheeler 1907). *Trachymyrmex pakawa* workers usually forage and arrive at the nest entrance singly; loose trails of four and six ants were observed late in the afternoon on only two occasions. At any time only about four ants can be seen in the general vicinity of the nest. Foragers are very shy and, when disturbed, they retreat inside the nest or feign death and drop to the ground if disturbed at some distance from the nest. This makes them difficult to detect in the field because they are easily scared. Apparently only a very small fraction of the nest´s population leaves the nest at any time to forage, at least during the day. It is not known if nocturnal foraging is significant. Regarding worker numbers in colonies, at least 250 workers emerged from the partially excavated nest by the spring at Lomas de Lourdes. The observed nest chamber was at about 50 cm deep in the spaces between rocks in unusually moist soil. There appeared to be only one queen in this nest.

The reddish detritus accumulations (exhausted fungal substrate) typical of several higher attines (Weber 1972, Sanchez-Peña 2005) are piled at 10–60 cm from the entrance hole and are conspicuous when present. The detritus accumulations of *T. pakawa* are flat, about 6 cm across. On slopes the detritus does not accumulate.

*Trachymyrmex pakawa* is a rather cryptic ant. At all locations it is never abundant and foraging workers seem to avoid open flat spaces and clearings, instead walking inconspicuously between vegetation and rocks. At La Estanzuela, Nuevo Leon, workers were noticeably more common at highest elevations (650 m), in the oak-pine forest, at the most mesic conditions observed for this ant.

Soil type (or lack of) might be a major, common factor regarding the distribution of *T. pakawa*. Excepting the lower altitude site at La Estanzuela, rocky, very shallow soils prevail at all locations, with large buried rocks, and always on slopes. Precipitation varies from 300 (UAAAN) to 600 mm (Estanzuela). However, drizzle and fog are frequent at Lourdes and UAAAN, and contribute an unmeasured amount of effective moisture.

This ant nests in the more mesic microhabitats (permanent and intermittent springs, creeks, or under and between large rocky outcrops) in a generally arid mountain range. The extant populations could be relicts of a more widespread distribution that contracted due to the post-glacial desertification process reducing the extension of woodlands and originating the Chihuahuan desert about 8000 years ago (Elias et al. 1995).

Although it is spread over an area covering a few hundred square kilometers, *T. pakawa* appears to have a discontinuous distribution in its range, and it is not abundant or common.

*Trachymyrmex pakawa* is not found in disturbed habitats. No population seems to have colonized agricultural areas (irrigated or dry land), or urban/landscaped areas in the neighboring cities (Monterrey and Saltillo) unlike other *Trachymyrmex* species. In this respect, this species differs markedly from geographically close North American species of *Trachymyrmex* that colonize (sometimes abundantly) gardens, yards, and landscaped areas in towns and cities, like the following: *T. smithi*, in northern Chihuahua (Ojinaga) and adjacent Presidio, Texas (SRSP unpublished observations); *T. septentrionalis*, in Austin, Texas (SRSP unpublished observations); and *T. turrifex*, in gardens in Pesqueria, Nuevo Leon, and in Matamoros, Tamaulipas, Mexico (Lower Rio Grande Valley); in this last case at more than 10 nest entrances/m^2^ (Sanchez-Peña 2010; see Rabeling et al. 2007). These apparently anthropo-tolerant *Trachymyrmex* populations live in deep, sandy, light-colored soils, unlike the thin, rocky, shallow limestone-based lithosols and steep alluvial soils near Monterrey and Saltillo colonized by *T. pakawa*. This spatial separation from humans might result from the strong observed preference of *T. pakawa* for rocky slopes and its avoidance of flat areas with deep soils.

Some of the collected specimens at La Estanzuela were conspicuously lighter than the ants from Saltillo and other sites; these are dark brown and initially they were confused with the even darker-colored *T. smithi*. Subsequent collections at La Estanzuela were more uniformly dark. It is possible that the lighter workers had emerged recently (callow workers). However, presumably callow workers from an excavated nest at Saltillo, covered with actinomycete growth (Cole 1952, Rabeling et al. 2007) possessed uniformly dark cuticle under the actinomycete layer. Recently emerged (callow) workers of *Trachymyrmex* spp. are commonly covered with growth of (potentially symbiotic) actinomycetes that give them a “sugary” appearance (Rabeling et al. 2007). The taxonomic relevance of the color differences described above is unknown.

Additional differences between *T. smithi* and *T. pakawa* include: in *T. smithi*, nests are often readily visible on flat soils in clearings, with conspicuous detritus piles nearby; nests can have a few hundred ants/nest; *T. smithi* is also somewhat aggressive and, when the nest or workers are disturbed, workers display aggressive behavior, and sometimes exit the nest resolutely, in small numbers, in an aggressive way (SRSP unpublished observations) unlike the very timid *T. pakawa*.

The discovery of *T. pakawa* indicates that the temperate Madrean ecoregion of the Sierra Madre Oriental might harbor additional interesting, undescribed arthropod taxa. This underscores the relevance of analysis and urgent conservation plans for these regions and biomes.

## Supplementary Material

XML Treatment for Trachymyrmex pakawa
